# Supplementary material for: Nanoscale resolution of microbial fiber degradation in action
Source: eLife. 2022 May 31;11:e76523. doi: 10.7554/eLife.76523 (PMC9191890; doi:10.7554/eLife.76523)
Supplement: Figure 2—figure supplement 2—source data 1. [file elife-76523-fig2-figsupp2-data1.zip › F2SD1.docx]

**Figure 2-figure supplement 2-Source Data 1 : Data collection and processing statistics of the cryo-EM structure**

| **Data collection** | |
| --- | --- |
| Microscope | FEI Titan Krios |
| Voltage (kV) | 300 |
| Camera | Gatan K2 Summit |
| Camera mode | Super-resolution |
| Energy Filter | Gatan postcolumn quantum energy filter (20 eV slit) |
| Nominal Magnification | 58’180 |
| Pixel Size (Å) | 0.4297 (in super-resolution)  0.8594 (for reconstruction) |
| Defocus range (μm) | -0.5 to -1.5 |
| Total electron dose (e/Å^2^) | 67 |
| Number of movies | ~2000 |
| **Data processing** | |
| Software | RELION 2.1 and RELION 3.0 |
| Initial number of particles | 1’331’906 |
| Final number of particles | 55’245 |
| Symmetry | C1 |
| Map resolution (Å)  FSC threshold | 3.38  0.143 |
| Map resolution range (Å) | 3.4 to 3.8 |
| Resolution of sharpened map (Å) | 3.38 |
| Resolution of unsharpened map (Å) | 3.44 |
| Map sharpening B-factor (Å^2^) | -111 |
| **Refinement** | |
| Refinement package | Phenix (Real-space refinement at 3.40Å) |
| Initial model used (PDB code) | 5YJ6 |
| Map-model CC | |
| CC_mask | 0.73 |
| CC_volume | 0.68 |
| CC_peaks | 0.56 |
| CC_box | 0.62 |
| Model composition | |
| Non-hydrogen atoms | 5180 |
| Protein residues | 636 |
| Ligands | 0 |
| *B* factors (Å2) (mean) | |
| Protein | 14.14 |
| R.M.S. deviations |  |
| Bond lengths (Å) | 0.004 (0) |
| Bond angles (°) | 0.616 (0) |
| Validation | |
| MolProbity score | 1.45 |
| Clashscore | 3.51 |
| Rotamer outliers (%) | 0.56 |
| Ramachandran plot | |
| Favored (%) | 95.58 |
| Allowed (%) | 4.26 |
| Outliers (%) | 0.16 |
| Cb boutliers (%) | 0 |
| CaBLAM outliers (%) | 2.06 |
